# Rising Cyclin-CDK Levels Order Cell Cycle Events

**DOI:** 10.1371/journal.pone.0020788

**Published:** 2011-06-10

**Authors:** Catherine Oikonomou, Frederick R. Cross

**Affiliations:** Laboratory of Cell Cycle Genetics, The Rockefeller University, New York, New York, United States of America; Duke University Medical Center, United States of America

## Abstract

**Background:**

Diverse mitotic events can be triggered in the correct order and time by a single cyclin-CDK. A single regulator could confer order and timing on multiple events if later events require higher cyclin-CDK than earlier events, so that gradually rising cyclin-CDK levels can sequentially trigger responsive events: the “quantitative model” of ordering.

**Methodology/Principal Findings:**

This ‘quantitative model’ makes predictions for the effect of locking cyclin at fixed levels for a protracted period: at low cyclin levels, early events should occur rapidly, while late events should be slow, defective, or highly variable (depending on threshold mechanism). We titrated the budding yeast mitotic cyclin Clb2 within its endogenous expression range to a stable, fixed level and measured time to occurrence of three mitotic events: growth depolarization, spindle formation, and spindle elongation, as a function of fixed Clb2 level. These events require increasingly more Clb2 according to their normal order of occurrence. Events occur efficiently and with low variability at fixed Clb2 levels similar to those observed when the events normally occur. A second prediction of the model is that increasing the rate of cyclin accumulation should globally advance timing of all events. Moderate (<2-fold) overexpression of Clb2 accelerates all events of mitosis, resulting in consistently rapid sequential cell cycles. However, this moderate overexpression also causes a significant frequency of premature mitoses leading to inviability, suggesting that Clb2 expression level is optimized to balance the fitness costs of variability and catastrophe.

**Conclusions/Significance:**

We conclude that mitotic events are regulated by discrete cyclin-CDK thresholds. These thresholds are sequentially triggered as cyclin increases, yielding reliable order and timing. In many biological processes a graded input must be translated into discrete outputs. In such systems, expression of the central regulator is likely to be tuned to an optimum level, as we observe here for Clb2.

## Introduction

In budding yeast, a single essential Cyclin Dependent Kinase (CDK) is alternately activated by nine cyclins to trigger the major events of the cell cycle. These cyclins are differentially expressed, inhibited, and degraded, and their temporal order of activity contributes to the ordering of cell cycle events [1], [2]. Within a given portion of the cell cycle, however, multiple events can be regulated by a single cyclin-CDK complex. Clb2, in the absence of the other mitotic cyclins, can promote all essential mitotic events with near-wild-type (WT) efficiency [3], [4] (Fig. S1). Moreover, this simplified system likely reflects the ancestral eukaryotic cell cycle control system, given strong evidence for gene and genome duplication events leading to the extant cyclin diversity [5], [6]. Importantly, even when driven by a single cyclin-CDK, mitotic events are temporally separated and exhibit a stereotyped order. Specifically, growth is depolarized, the spindle forms, and then elongates. In the absence of unique cyclin-CDK activators, what determines the order and timing of such mitotic events?

Checkpoints [7] that halt mitotic progression are non-essential in budding yeast and largely do not contribute to normal cell cycle timing [8], [9]. Mechanistic coupling, in which a later event is structurally dependent upon completion of an earlier event [10], is limited to events that involve the same structure, such as spindle formation and elongation. Alternatively, later events may require higher CDK activity levels than earlier events, a possibility known as the “quantitative model” of ordering [11].

Recent work using a monomeric engineered cyclin-CDK in fission yeast has provided evidence that the “quantitative model” promotes the ordering of S-phase and mitosis in that organism. The cyclin-CDK was engineered so that it could be inhibited by an ATP analog. The authors found concentrations of analog that permitted S-phase but not mitosis, suggesting a higher cyclin-CDK threshold for the later phase [12]. Two recent studies of cyclinB1-CDK1 activation dynamics in HeLa cells and extracts have also provided support for this quantitative cyclin-CDK activity level model. It was shown that in prophase, later events require more cyclinB1-CDK1 activity than earlier events [13]. It was also shown, *in vitro*, that later-acting cyclinB1-CDK1 substrates require higher levels of cyclinB1 for phosphorylation than earlier-acting substrates [14]. Mitosis in budding yeast provides an ideal *in vivo*, organismal system to expand these studies. Clb2 can control all mitotic events, and the Wee1 and Cdc25 positive feedback loops that control CDK activation in higher eukaryotes are not essential in budding yeast [15], greatly simplifying the experimental system. In addition, genetic tools allow us to titrate cyclin levels, *in vivo*, within the endogenous range. This titration, in combination with single-cell timelapse imaging, allows us to quantitatively measure and compare thresholds for cyclin-CDK control of individual mitotic events.

To determine whether the timing of mitotic events is normally controlled by Clb2-CDK level, we quantitatively measured the Clb2 requirements for three mitotic events – growth depolarization, spindle formation, and spindle elongation. We address whether these events exhibit thresholds in response, compare Clb2 requirements to the levels of Clb2 in freely cycling cells when these events occur, and analyze the effects of premature Clb2 accumulation on the timing of these events.

## Results

### A System to Measure Mitotic Cyclin-CDK Requirements

**Figure 1:**
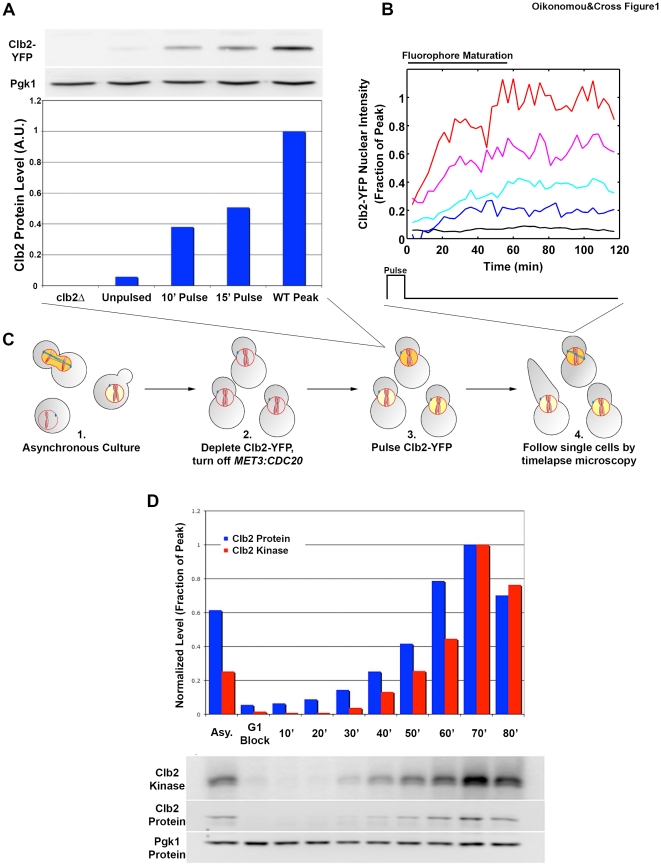
Experimental system to measure Clb2-CDK requirements. **A** Clb2-YFP protein from populations of cells pulsed with 5mM DOC for 10′ or 15′ compared to Clb2-YFP protein levels from *clb2Δ* cells, unpulsed cells of the experimental strain, and the peak expression level of *clb1,3,4Δ* cells synchronized with alpha factor and released. Below, Clb2-YFP levels are normalized to Pgk1 (which is not cell-cycle regulated) and expressed as a fraction of the peak value. **B** Mean Clb2-YFP intensity values in single cells following a pulse of DOC. Each colored trace represents a different cell. Approximately the first 40 minutes correspond to the maturation time of the fluorescent protein. **C** Schematic of experimental protocol for assaying mitotic Clb-CDK requirements for mitotic events. *clb1,3,4Δ* cells expressing Clb2-YFP in response to DOC are grown asynchronously in the presence of DOC (1). DOC is washed out, arresting cells through Clb2 depletion with replicated DNA and unseparated SPBs (2). Methionine is added to turn off *CDC20* expression (and thus Clb2-YFP degradation). Cells are given a pulse of DOC (3) and followed by timelapse microscopy (4). Execution of mitotic events is correlated with stable Clb2-YFP level in single cells. **D** Clb2 is normally active for at least 40 minutes per cell cycle. *clb1,3,4Δ* cells were synchronized with alpha factor and released. Clb2 protein and kinase activity levels were assayed as shown. Quantification of blots is shown, with values normalized to Pgk1 and expressed as a fraction of the peak value at 70′.

There are four, largely redundant, mitotic cyclins in budding yeast: Clb1-4. Clb2, in the absence of the other three, can successfully promote all essential mitotic events, with near-WT timing (Fig. S1) [3], [4]. We wanted to develop a system to measure the Clb2-CDK requirements for individual mitotic events. To do this, we constructed a *clb1,3,4Δ GALL:CLB2* strain, in which *CLB2* is the sole source of mitotic cyclin, and is under the control of an attenuated galactose-inducible (*GALL)* promoter [16], [17]. We added a construct encoding a fusion of *GAL4* (the activator of the *GALL* promoter) with a mammalian mineralocorticoid receptor [18]. *CLB2* expression was now dependent upon the presence of an exogenous hormone, deoxycorticosterone (DOC), so experiments could be carried out in a single carbon source, and the expression level achieved was within the physiological range for *CLB2* (Fig. 1A) [19]. To measure the Clb2 concentration in single cells, we assayed the fluorescence intensity of YFP-tagged Clb2, which is fully functional (B. Drapkin, personal communication; data not shown). Clb2 is predominantly localized to the nucleus [20], and nuclear size is tightly correlated with overall cell size [21], so we used a histone H2B-mCherry fusion to mark the nucleus, and measured the mean YFP intensity within this mask (with unlabeled cell background subtracted) to estimate total Clb2-YFP concentration per cell. To allow cells to be incubated for long periods with a titrated level of Clb2, we prevented its degradation by turning off the Anaphase Promoting Complex (APC) activator *CDC20* using a methionine-repressible promoter (*MET3*). Clb2-YFP pulses induced in the presence of methionine (and therefore in the absence of *CDC20* expression) were stable for at least two hours (Fig. 1B). The overall experimental protocol was the following (shown in Fig. 1C): 1. proliferate cells in the presence of DOC; 2. deplete Clb2-YFP by washing out the hormone, arresting cells prior to mitosis; deplete Cdc20 by adding methionine to the media (“block”); 3. give a pulse of Clb2-YFP by adding DOC and subsequently washing it out (“induce”); 4. use timelapse microscopy to correlate cell fate with mean background-subtracted nuclear Clb2-YFP intensity in single cells, normalized to peak Clb2 expression in cycling cells [19]. Previous studies have validated this quantification and shown the ‘proportion of peak’ units to have physiological meaning [19], [22].

Clb2 was present at low but detectable levels in blocked, uninduced cells. This may reflect inheritance of Clb2 from the cell cycle before the block, or basal expression from the *GALL:CLB2* construct. In any event, this level of Clb2 is insufficient to drive entry into mitosis, as >90% of these cells stably failed to make a spindle for at least two hours in the absence of Clb2 induction.

We are using Clb2 protein level as a surrogate for Clb2-CDK activity. However, the Clb2-CDK-inhibitory kinase Swe1 is present and potentially active in these cells. On the other hand, Swe1 activity might not be expected to be high, since these cells are budded, so the ‘morphogenetic checkpoint’ should not activate Swe1 [23]. To directly address the potential regulation of Clb2-CDK activity by Swe1, we measured Clb2-CDK kinase activity throughout the experimental protocol, using *SWE1* and *swe1Δ* cells. We found that the presence of Swe1 approximately halves the kinase activity of Clb2-CDK for about the first 30 minutes following the expression pulse (Fig. S2A). To compare this to the situation in normally cycling cells, we used alpha factor to synchronize WT and *swe1Δ* strains in G1, and measured the Clb2-CDK kinase activity following release. We observed that Swe1 lowers Clb2-CDK kinase activity by a similar factor, again for approximately the first 30 minutes of Clb2 expression, corresponding to early mitosis (Fig. S2B). This indicates that our expression system faithfully recreates physiological conditions of transient kinase inhibition. Since the kinase assay is a bulk population measurement, we do not know if the early 2-fold reduction in Clb2 kinase is uniform across the population. We return to this issue below. To keep the experimental system as close as possible to physiological conditions in the cell, we conducted most subsequent experiments in *SWE1* strains, with Clb2 levels reasonably reflecting Clb2-associated kinase activity.

Clb2-CDK can also be stoichiometrically inhibited by Sic1. However, Sic1 is likely absent in the Clb-depleted cells in our protocol, since they express a high level of the G1 cyclin Cln2 [24], which promotes efficient Sic1 degradation [25]. At later times in the *SWE1* timecourses and throughout the *swe1Δ* timecourses, the activity of Clb2-associated kinase closely paralleled Clb2 protein levels. Therefore, it is unlikely that Sic1 inhibition is significantly regulating Clb2-CDK activity in these experiments. Sic1 is completely degraded by mid-cell-cycle [26] and if it were inhibiting Clb2-Cdk earlier, we should observe a change in specific activity later when inhibition is lifted.

### Ordering of Mitotic Events by Cyclin-CDK Level

Using alpha-factor synchronization of a *clb1,3,4Δ* strain, we determined that Clb2 protein level, and associated Clb2-CDK kinase activity, ramped up over a period of about 40 minutes (Fig. 1D). Thus, if different activity levels promoted different events, these events could be significantly separated in time. We chose to measure the Clb2 requirement for three temporally separated mitotic events: depolarization of growth, spindle formation, and spindle elongation. In *CLB2^WT^* cells synchronized in G1 with alpha factor and released, growth depolarization (see Materials and Methods for details of measurement) occurs, on average, 20 minutes before spindle formation (Fig. S3); spindle formation occurs approximately 10 minutes before spindle elongation (anaphase). Importantly, all of these events occur as Clb2 levels are steadily increasing. Therefore, if the different events are induced by sequentially higher Clb2 levels, the gradual rise of Clb2 could in principle provide a simple ordering mechanism.

### Growth depolarization: regulation of an autonomous oscillator

**Figure 2:**
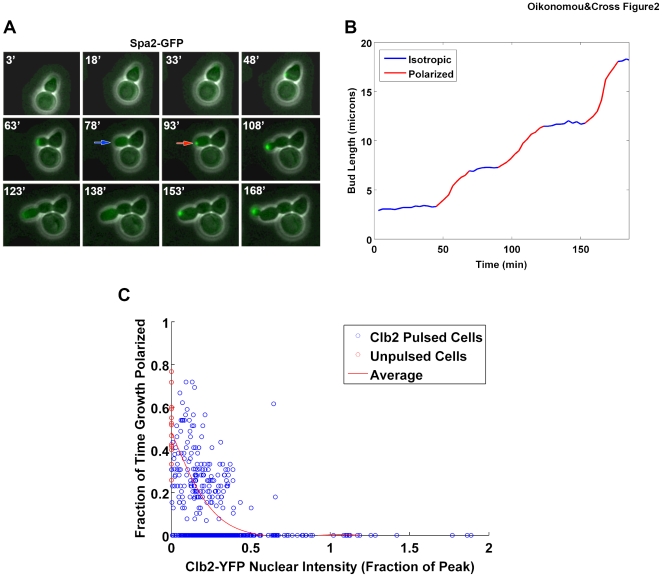
Clb2-CDK control of growth depolarization. **A** Images from a single cell of the experimental strain arrested by washout of DOC (unpulsed) and imaged by timelapse microscopy for the polarisome protein Spa2-GFP. Images are overlays of phase contrast and CFP composites of three z-stacks, 0.5um apart. Red arrow indicates localization, and blue arrow delocalization. **B** Bud length as a function of time for the cell shown in A. Color-coding as in A (red, Spa2-GFP localized; blue, delocalized). **C** Fraction of time single cells exhibit polarized growth (measured by rate of increase of bud length) as a function of Clb2-YFP level, following a pulse of Clb2-YFP. Red points indicate unpulsed cells, blue points indicate cells that received a pulse of Clb2-YFP, and the red line indicates the average fraction for binned Clb2-YFP levels. N = 538.

In budding yeast, cellular growth is polarized early in the cell cycle. At the time of bud initiation, all growth is focused to the bud tip, resulting in initial formation of an elongated bud with actin polarized to the bud tip (‘polarized growth’). Later in the cell cycle, bud growth and actin filaments are depolarized in a Clb-CDK dependent manner, resulting in rounded bud growth (‘isotropic growth’) [27]. We used two metrics to assay polarized growth: localization of the Spa2 ‘polarisome’ component to the bud tip [28], and the rate of increase of bud length (which is significantly higher during polarized growth). Interestingly, in the absence of mitotic Clb-CDK activity, we observed that growth was not continuously polarized but rather exhibited alternating cycles of polarized and depolarized growth, each lasting approximately 40 minutes (Fig. 2A,B; Video S1) (polarized, 40.1±15.8′; depolarized, 39.0±13.0′). Due to the tight correlation of our two metrics, we assayed only bud length increase in subsequent experiments. These findings are consistent with previous findings suggesting an intrinsic mitotic cyclin-independent oscillator controlling cell polarization and budding [29]. We pulsed cells with Clb2-YFP and tracked polarized growth to determine the response of this polarized growth oscillator as a function of Clb2-YFP level. Although there is significant variability in the measured response to low Clb2 levels in this assay, we cannot reliably assign it to either experimental error or true variability in the biological response because polarized growth is periodic in the absence of any Clb2. Since we do not know where in the polarized growth cycle any individual cell was at the time of the Clb2-YFP pulse, considerable variability in the assay is expected, even if the real response to a given Clb2 level were completely deterministic. However, we clearly saw that Clb2-YFP exerted dose-dependent effects: the higher the Clb2-YFP level, the less time a cell exhibited polarized growth (Fig. 2C). Clb2-CDK appeared to decrease both the frequency of the cycle, and the duration of the polarized period within each cycle. Remarkably little Clb2-YFP was sufficient to depolarize growth; 50% of cells completely depolarized their growth with 0.12 peak Clb2-YFP. In synchronized WT cells, depolarization occurs when cells contain approximately 0.3 peak Clb2-YFP (Figs. 1D, S3); consistently, in Clb2-pulsed cells, the same level induces 75% of cells to completely depolarize their growth.

Recently, we presented evidence that a free-running oscillator controlling nucleolar localization of the Cdc14 phosphatase was entrained to occur once per cell cycle by mitotic cyclin-CDK-dependent modulation of its frequency through a phase-locking mechanism [22]. The finding that Clb2-CDK activity could also modulate the frequency of the bud growth oscillator suggested that phase-locking might similarly constrain this oscillator to once per cell cycle. Indeed, a simple phase-locking model [22] can be modified to incorporate the polarization oscillator we observe here. Using parameters approximated from the present experimental results, the model suggests that levels and timing of Clb2 accumulation are sufficient to effectively phase-lock the bud growth oscillator to once per cell cycle (Fig. S4).

### Spindle formation

**Figure 3:**
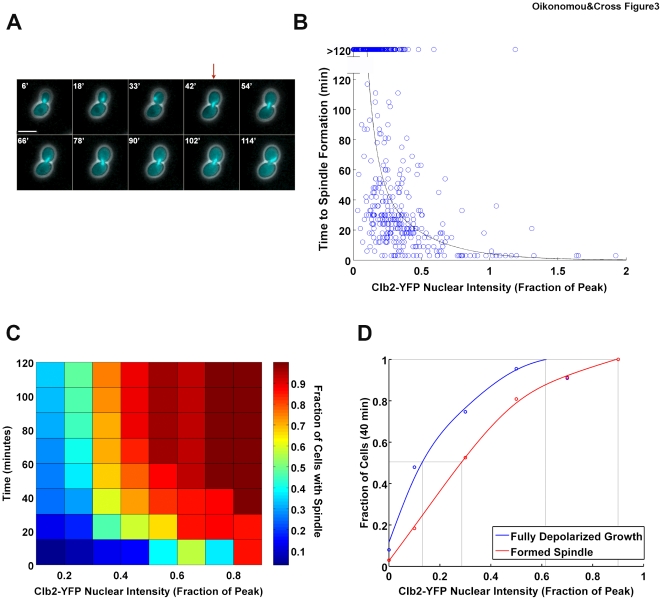
Clb2-CDK requirement for spindle formation. **A** Example of a cell exhibiting spindle formation (red arrow) during timelapse imaging, following a pulse of Clb2-YFP. Scale bar: 5 µm. Images are composites of phase contrast and CFP channels. **B** Time of spindle formation in single cells following a pulse of Clb2-YFP. Points greater than 120′ indicate cells that did not form a spindle within the two-hour time frame of the movie. The black line indicates the median time of spindle formation at binned Clb2-YFP levels. N = 496. **C** Heat map using data shown in B. Colors reflect the fraction of cells that formed a spindle by the indicated time at the indicated level of Clb2-YFP. **D** Dose response curves, calculated as follows: for growth depolarization, we calculated the fraction of cells with a given binned Clb2-YFP value which completely depolarized their growth (fraction of time  = 0); for spindle formation, we calculated the fraction of cells with a given binned Clb2-YFP value which formed a spindle within 40′. We chose 40′ as this is the average depolarized growth cycle time (and thus the limit of our ability to detect depolarization) as well as the amount of time a cell is normally exposed to Clb2. Grey lines indicate 50% and 100%.

We next measured the amount of Clb2-YFP required for spindle formation, assayed with fluorescently-labeled tubulin (Tub1-CFP) (Fig. 3A; Video S2). Spindle formation exhibited a steep acceleration in response to increasing Clb2-YFP levels (Fig. 3B,C). Interestingly, while cells with low Clb2 levels sometimes waited several hours before forming a spindle, the actual event (marked by conversion of a tubulin ‘dot’ to a ‘bar’ of stable length) almost invariably occurred within 6 minutes, at all Clb2 levels.

To confirm that Tub1-CFP was accurately reporting spindles, we repeated the experiment with a strain containing a fluorescently-labeled spindle pole body (SPB) component (Spc29-CFP). As we could not reliably follow SPBs using timelapse microscopy (due to their small size and mobility), we fixed cells one hour after a pulse of Clb2-YFP and observed SPB separation (two vs. one dots of Spc29-CFP) as a function of Clb2 dosage. These data were consistent with the Tub1-CFP timelapse data at the equivalent timepoint (Fig. S5A). Thus, the Tub1-CFP assay faithfully detects spindle formation.

We also determined the endogenous Clb2 level that allows spindle formation by synchronizing *clb1,3,4Δ CLB2-YFP* cells with alpha factor, turning off *MET3:CDC20* by adding methionine (to inhibit Clb2-YFP degradation), and adding cycloheximide around 50 minutes after release to allow YFP maturation without further protein synthesis. One hour after cycloheximide addition, we fixed the cells and correlated Clb2-YFP level with spindle state. In control cultures, about 50% of the cells already contained spindles 50 minutes after alpha factor release. Since this proportion was similar to that observed after one hour in cycloheximide, most of the spindles had most likely already formed at the time of cycloheximide addition. Therefore, we can reasonably extrapolate the final Clb2-YFP fluorescence level as the level at the time of spindle formation. We observed spindles only in cells that contained Clb2 levels similar to, or higher than, the threshold level measured in the pulsed cells, consistent with physiological relevance of these measurements (Fig. S5B).

One notable feature of the Clb2 dose response data is its variability. We can reliably measure the timing of spindle formation within two frames. We can also accurately measure the Clb2-YFP level in single cells due to careful normalization, as well as to the stability of the fluorescent protein. This indicates that the variability is not experimental, but rather reflects the biological system. The stoichiometry of phosphorylation targets for spindle formation is not known, but it may be low. For example, for the putative Clb-CDK target Sfi1, it is estimated that there are only a few molecules in the SPB bridge [30], [31]. We constructed a simple simulation in which separate targets must be stochastically and independently phosphorylated by Clb2-CDK, in the presence of countervailing phosphatase activity, in order to trigger spindle formation. Low numbers of targets led to a prediction of a highly variable time to spindle formation with low Clb2 levels, but rapid and efficient spindle formation at high Clb2 levels, qualitatively matching our experimental results (Fig. S6).

Spindle formation required significantly more Clb2-YFP than growth depolarization (Fig. 3D). 0.12 peak Clb2-YFP was sufficient to induce depolarized growth in 50% of cells; 0.27 peak Clb2-YFP was required to induce spindle formation in the same fraction. Interestingly, in our experimental system, we occasionally observed cells with sustained, low Clb2-YFP levels that eventually formed a spindle without depolarizing their growth. This is possible because of the variability in response noted above; this uncoupling indicates that there is no intrinsic block to misordering of these events. A further implication is that response variability is probably not due to cell-wide variability in Clb2-CDK activity, but rather to stochastic variation in response to a given Clb2-Cdk level. However, on average, there is a steep inverse relationship between the time cells take to form a spindle and their Clb2 level. The average time to form a spindle at low Clb2 is significantly greater than the total cell cycle time, compared to minutes at high Clb2. Therefore, despite the high degree of noise evident in the system at low Clb2 levels, the rate of increase of Clb2 in a normal cell cycle results in a prediction of smooth and reliable timing, with spindle formation following depolarization by a significant interval, in the large majority of cell cycles. This prediction has been confirmed by simulation (data not shown).

In WT cells, Clb3 and Clb4 contribute to spindle formation [4], [32], but not to growth depolarization [27]. Our simplified system removes any contribution from functional specialization of mitotic cyclins. The reality of the system likely lies between the extreme models of accumulation of bulk undifferentiated cyclin, simulated here, and complete functional specialization of differentially-expressed cyclins. It is therefore important to note that the timing of Clb3 and Clb4 accumulation likely contributes to the relative timing of spindle formation in an unperturbed cell cycle.

### Swe1 modulates the timing of early mitotic events

**Figure 4:**
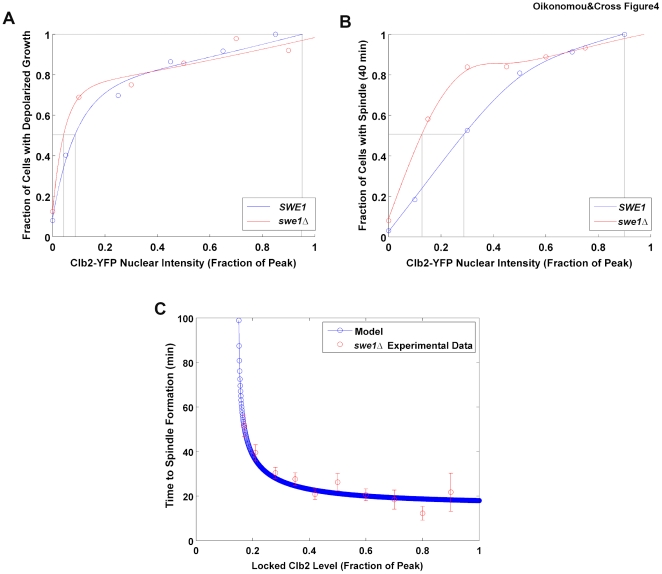
Swe1 controls the timing of early mitotic events. **A** Dose response curves for complete growth depolarization (fraction of time growth polarized  = 0) for *SWE1^WT^* (blue) and *swe1Δ* (red) strains. N = 472. Grey lines indicate 50% and 100%. **B** Dose response curves for spindle formation within 40′ for strains with (blue) or without (red) *SWE1*. N = 472. Grey lines indicate 50% and 100%. **C** Simulation of quantitative model for spindle formation with fixed Clb2 levels. Time of spindle formation is shown as a function of Clb2 level for the model (blue). Red points indicate the mean time of spindle formation for binned Clb2-YFP nuclear intensity levels from experimental Clb2-YFP pulse data (*Δswe1* strain). Error bars indicate SEM.

This model implies that factors altering the rate of accumulation of cyclin-CDK activity should modulate the timing of events. Swe1 is not essential for normal cell cycle progression in yeast, but its absence results in a two-fold increase in Clb2-associated kinase activity in early mitosis (Fig. S2), and spindle formation may be accelerated in *swe1Δ* strains [33] (but see also [34]). Consistent with this, we found that the threshold Clb2 requirement for both growth depolarization and spindle formation was lowered by about two-fold in a *swe1Δ* background (Fig. 4A,B). This coincidence between the two-fold threshold reduction measured in individual cells, and the two-fold reduction in kinase activity measured in a bulk population (see above) suggests that the two-fold kinase reduction may actually pertain to most individual cells, rather than reflecting, for example, a 1∶1 mix of fully inhibited and uninhibited cells. Thus, Swe1 seems to act as a regulatory input to tune the timing of early mitotic events. This correlation supports the idea that the measured thresholds at locked Clb2 levels are relevant to timing in the free-running cell cycle.

### Testing a mathematical cell cycle model

We used our measurement of the Clb2-CDK requirement for spindle formation to test a published quantitative Ordinary Differential Equation (ODE) model of the budding yeast cell cycle [35]. We isolated the module associated with spindle formation and simulated its response to various stable Clb2 concentrations, mimicking our experimental system. As shown in Figure 4C, we observed a threshold corresponding to about 15% of the peak Clb2 level, about half the value we observed experimentally. Interestingly, this is very close to the threshold we observed in a *swe1Δ* strain. In fact, this is the appropriate comparison, as the computational model [35] did not include Swe1 inhibition of Clb2-CDK. Furthermore, at critical values of Clb2 (0.15–0.2 peak), the time of spindle formation predicted by the model increases dramatically from the normal ∼20 minutes to nearly two hours. This is in accordance with our experimental results (Fig. 4C), suggesting that the model has an appropriate titration of Clb2 level to spindle formation, despite the lack of any mechanistic detail in this part of the model's construction.

### Spindle elongation/anaphase

**Figure 5:**
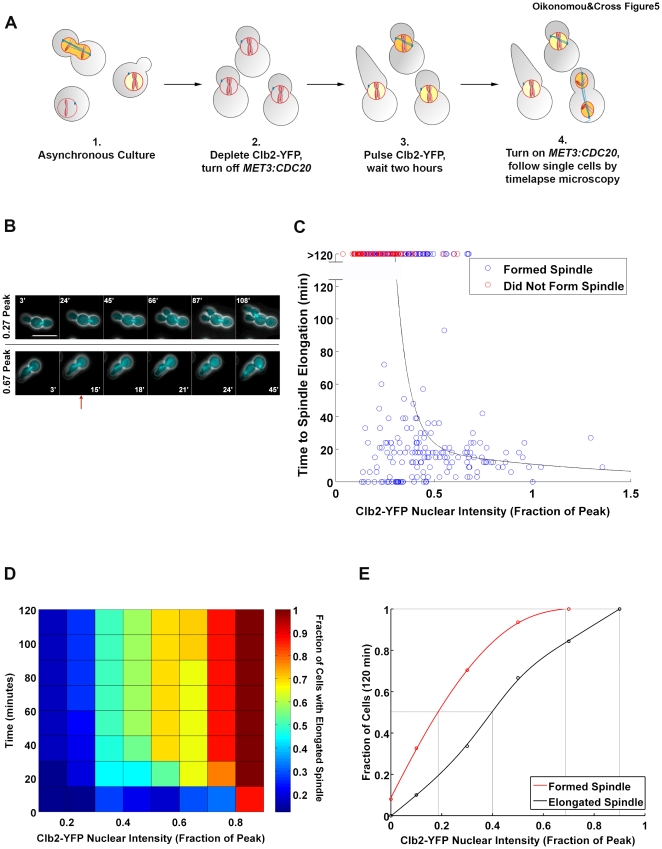
Clb2-CDK requirement for spindle elongation. **A** Schematic of the experimental protocol for assaying the Clb2-YFP requirement for anaphase. *clb1,3,4Δ* cells expressing Clb2-YFP in response to DOC (1) are arrested by DOC washout and addition of methionine (2), cells are exposed to a pulse of DOC and given 2 hours to form spindles (3), methionine is washed out and cells are followed by timelapse microscopy (4). **B** A representative cell that does not elongate its spindle is shown at top, and a cell that does (red arrow) is shown at bottom. The Clb2-YFP mean nuclear intensity for each cell is shown to the left. Scale bar: 5 µm. **C** Time of spindle elongation (assayed by Tub1-CFP) in single cells of the experimental strain following a pulse of Clb2-YFP. Blue points greater than 120′ indicate cells which had short spindles that did not elongate; red points indicate cells that never formed spindles. N = 239. **D** Heat map showing the fraction of cells that elongated their spindle by the indicated time at the indicated level of Clb2-YFP. **E** Dose response curves for spindle formation and spindle elongation within 120′. Grey lines indicate 50% and 100%. Note difference in time of assay compared to Figure 3D.

Finally, we examined a late step in cell cycle progression: spindle elongation or anaphase. Clb2-CDK promotes anaphase through at least two pathways. First, Clb2-CDK activates APC^Cdc20^, which drives separation of sister chromatids [36], [37]; Clb2-CDK also promotes spindle elongation through an APC-independent mechanism [38]. To measure the Clb2 requirement for anaphase, we depleted Clb2-YFP and Cdc20 as before, pulsed cells with Clb2-YFP, allowed two hours for spindle formation, then washed out the methionine in the medium to allow Cdc20 reaccumulation and followed single cells by timelapse microscopy (Fig. 5A,B; Videos S3, S4). A caveat of this experimental approach is that Cdc20 accumulation is normally cell cycle regulated; by expressing Cdc20 from an exogenous promoter, we bypass this control. This is an experimental necessity with available reagents. Spindle elongation (assayed by Tub1-CFP) occurred with increasing efficiency as a function of pre-anaphase Clb2-YFP level (Clb2-YFP was degraded in these cells as anaphase proceeded) (Fig. 5C,D). We rarely observed cells with high Clb2-YFP levels, presumably because these cells rapidly entered anaphase and initiated Clb2-YFP degradation during sample preparation (≤10 minutes). Importantly, not all cells that formed spindles subsequently elongated them (Fig. 5B top and blue points above 120′ in Fig. 5C), and these cells contained, on average, lower Clb2-YFP levels than those which did elongate their spindles. This indicates that these two events are not ordered solely by mechanistic coupling.

We compared the Clb2 requirements for spindle formation and elongation by calculating the fraction of cells with a given Clb2-YFP level that executed each event within 120 minutes (Fig. 5E). We chose 120 minutes as this is the time given cells in our assay to form spindles before restoring Cdc20 and allowing spindle elongation. As in the comparison between growth depolarization and spindle formation, the later event (spindle elongation) required significantly more Clb2 than the earlier event (spindle formation). These results are consistent with increasing cyclin-CDK levels triggering cell cycle events in a temporal order.

### Clb2 is Rate-limiting for Mitosis

**Figure 6:**
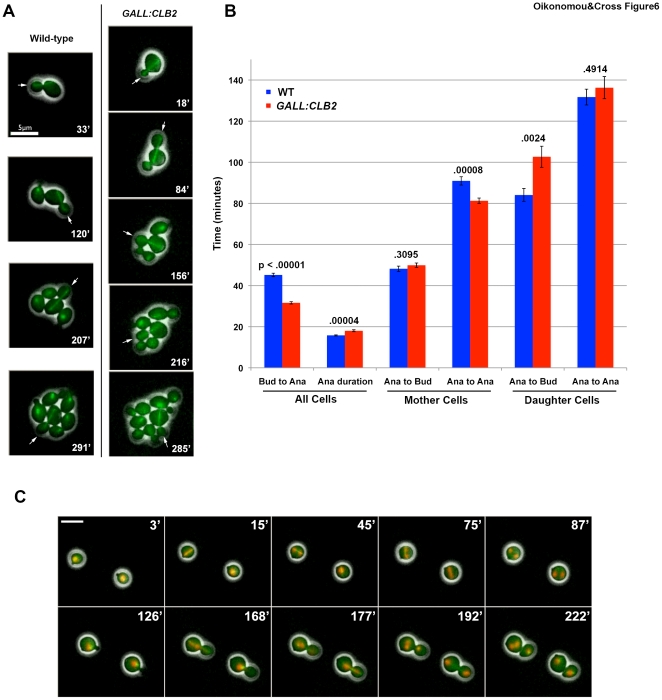
Increased Clb2 accelerates cell cycle timing. **A** *CLB ^WT^* and *GALL-CLB2* cells containing fluorescently-labeled tubulin (Tub1-GFP) were grown in galactose-containing media and followed by timelapse microscopy. Representative images of mother cells undergoing sequential anaphases (arrows) are shown (left column, WT; right column, *GALL:CLB2*), with time indicated in the bottom right-hand corner of each image. **B** Timing of *CLB ^WT^* (blue) and *GALL-CLB2* (red) cells cycling in galactose-containing media. The lengths of the indicated intervals were scored from phase contrast and Tub1-GFP images. Bud: bud formation; Ana: onset of spindle elongation; Ana duration: total time with elongated spindle. Mean ± SEM is shown, and numbers above the bars indicate p-values from a two-tailed two-sample t-test. **C** Timelapse images of two cells exhibiting premature anaphase. Tub1-GFP marks spindles; Htb2-mCherry marks nuclei. Images are composites of phase-contrast, GFP, and Texas Red channels.

If mitotic cyclin-CDK levels normally order mitotic events, it might be expected that increasing the cyclin concentration should accelerate mitosis. To test this, we replaced the endogenous *CLB2* in an otherwise *CLB^WT^* strain with *CLB2* under the control of the *GALL* promoter, which, when activated with galactose, results in Clb2 protein levels a few times higher than the endogenous level as measured in asynchronous cells. Using timelapse microscopy with fluorescently-labeled tubulin (*TUB1-GFP)* to monitor anaphase in cells freely cycling in galactose, we observed a shortened budded period (time between budding and anaphase), as well as significant acceleration of the overall cell cycle (Fig. 6A,B). *GALL:CLB2* mother cells took, on average, 81 minutes to progress from one anaphase to the next, while WT cells took an average of 91 minutes. These accelerated cell cycles were observed repeatedly in individual cells; a short cell cycle was not followed by a long one. Therefore, the intrinsic cell cycle frequency was significantly increased by *CLB2* overexpression.

Despite accelerated cell cycles in mother cells, the normal coupling of growth and division seems to remain intact in *GALL:CLB2* cells since the cycle time was decreased specifically through acceleration of the budding-to-anaphase time; anaphase to subsequent budding time is essentially unchanged in *GALL:CLB2* mothers. Coordination of growth and division in budding yeast is primarily effected by regulation of the daughter cell division-to-bud interval (essentially pre-Start [39]). Since the budded interval is shorter in *GALL:CLB2* cells, the size of the daughter at division is expected to be smaller, which we observed (∼20% smaller cross-sectional area of *GALL:CLB2* daughters compared to WT). The longer G1 interval in *GALL:CLB2* cells is thus a predicted consequence of the smaller daughter size.

By this reasoning, the overall doubling time of WT and *GALL:CLB2* cells should both be the same as the biomass doubling time (which we assume is unlikely to change significantly due to Clb2 overexpression). However, we observed that a WT population doubles ∼7% faster than a *GALL:CLB2* population (WT doubling time, 101±2 minutes; *GALL:CLB2*, 108±1 minutes). What is the nature of this fitness defect? In timelapse imaging, we observed a curious phenotype in approximately 6% of *GALL:CLB2* cell cycles. Cells with no or very small buds elongated their spindles and completed nuclear division within the mother cell. Following this inappropriate anaphase (which lasted approximately 10 minutes longer than normal), SPBs and nuclei often coalesced, the cells rebudded more or less on time, and completed another cell cycle. Both the mothers and daughters of this division, however, were aberrant, completing no more than one subsequent division before arresting, and often containing multiple nuclei (Fig. 6C; Video S5). The resulting loss of about 6% of the *GALL-CLB2* cells from the cycling population accounts for the reduction in population doubling time described above, and clearly represents a cost to Clb2-overexpressing cells.

**Figure 7:**
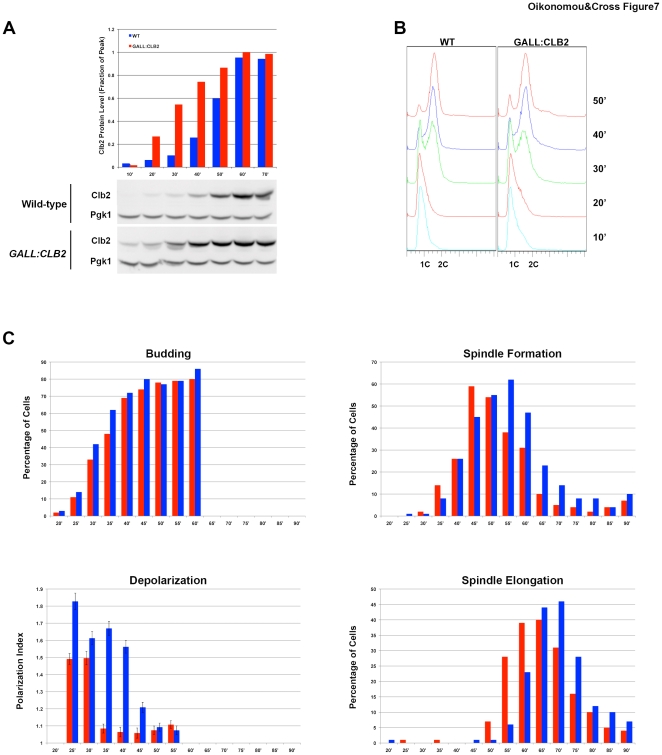
Clb2 is rate-limiting for mitotic events. **A** Clb2 and Pgk1 protein levels for WT (blue) and *GALL:CLB2* (red) strains at indicated time points after release from alpha factor. Quantification above shows Clb2 levels normalized to Pgk1 and expressed as a fraction of *GALL:CLB2* peak at 60′. **B** DNA content of the two strains at the indicated timepoints following release, analyzed by FACS. **C** Budding fraction, polarization index (values around 1 indicate depolarized growth; values >1 indicate polarized growth), metaphase fraction, and anaphase fraction of WT (blue) and *GALL-CLB2* (red) cells.

To measure acceleration of individual mitotic events by Clb2 overexpression, we synchronized *GALL:CLB2* and *CLB2^WT^* cells with alpha-factor and fixed samples for imaging every five minutes following release. Clb2 protein levels started very low in both cultures (Fig. 7A), probably due to efficient Cdh1-mediated Clb2 degradation at the alpha factor block. In *GALL:CLB2* cells, Clb2 levels rose faster than in WT, but attained a similar peak level before degradation in mitosis in both cultures. Thus, this experiment provides a clear system to analyze the effects of early expression (*without* significant overexpression) of peak Clb2 levels.

Budding and DNA replication occurred at similar times post-release in *GALL:CLB2* and WT cells (Fig. 7B,C). However, metaphase was slightly accelerated (5 to 10 minutes), and growth depolarization and anaphase significantly accelerated (at least 10 minutes) in *GALL:CLB2* cells. This shift is similar to the shift in timing of Clb2 accumulation, supporting the model that rising Clb2 directly controls the timing of these events. The time of budding is largely controlled by G1 cyclin accumulation [39], and DNA replication is similarly Clb2-independent, relying on the S-phase cyclins Clb5 and 6. These events therefore serve as independent markers of release from the alpha factor block, showing that acceleration of mitotic event timing is specifically due to premature Clb2 accumulation within the progressing cell cycle.

The observation of an accelerated switch to depolarized growth is consistent with previous work in which Clb2 was expressed from the *GAL1* promoter. In response to *GAL1:CLB2* expression, the fraction of buds exhibiting polarized growth was lower than in WT cells, suggesting an earlier switch to depolarized growth [27]. This result is somewhat complicated by the fact that such Clb2 overexpression delays cells prior to mitotic exit, at a stage of depolarized bud growth, and this mitotic delay would decrease the fraction of cells exhibiting polarized growth even if the switch was not accelerated. The results here provide an unambiguous demonstration that shifting Clb2 expression to earlier in the cell cycle, without overall gross overexpression, does indeed strongly accelerate the switch to depolarized growth.

Other previous work [40] suggested that high Clb2 expression did not accelerate mitotic events. The discrepancy may be accounted for by the very high overexpression in that work, in which 8 or more copies of *GAL1:CLB2* were used. This likely results in ∼80-fold higher Clb2 than in our experiments, since the *GAL1* promoter is about ten times the strength of the *GALL* promoter [16]. This level of Clb2 is lethal, and sufficient to lead to strong competition between Clb2 and other cyclins for available CDK [9], plausibly leading to multiple indirect effects that should not interfere with our analysis. We were also able to assay single cells in un- or minimally-perturbed cycling conditions, providing greater accuracy.

## Discussion

We describe here an *in vivo* system for testing endogenous cyclin-CDK requirements for physiological mitotic events. Our results are consistent with recent work in mammalian systems showing that increasing mitotic cyclin-CDK levels may order mitotic events, which suggests that this ordering mechanism is fundamental and conserved across eukaryotes [13], [14]. In addition, recent work using a monomeric engineered cyclin-CDK in fission yeast suggests that a similar mechanism controls the relative timing of S-phase and mitosis in that organism [12]. The work presented here extends previous results through rigorous quantitative tests, titrating cyclin levels within the endogenous range, in single cells, and assaying events spread across mitosis and involving multiple targets and biological systems.

While key events in the cell cycle may be triggered in a probabilistic manner by cyclin-CDK, and thus can occur stochastically in reverse order if CDK activity is held at a sustained low level, the speed of cyclin-CDK rise in a normal cell cycle, combined with the steepness of the Clb2 response thresholds, provides reliable, reproducible timing of these events in almost all cell cycles. Our work also confirms a key prediction of this ordering mechanism: increasing mitotic cyclin-CDK above the endogenous level results in faster cycling over multiple cell cycles.

The requirements for Clb2-CDK that we observe in our experiments are below peak Clb2 levels. Spindle elongation, the highest threshold, is reliably triggered by 0.9 peak Clb2 and in WT cells, Clbs1,3, and 4 contribute to mitosis as well, with a combined protein level estimated at a few times that of Clb2 alone [9], [41]. Thus, the cell appears to synthesize more mitotic cyclin than is strictly necessary. What is the advantage of producing excess cyclin? One explanation may lie in the kinetics of these events; as we observed for spindle formation and elongation, higher cyclin levels may ensure more efficient execution and buffer the inherent variability in the system. Additionally, we observed a high degree of variability among cycling cells in the peak Clb2 level reached during a given cycle; excess cyclin production may ensure that even low-expressing cells make enough cyclin to get through mitosis efficiently.

At the same time, very moderate overexpression of Clb2 above the wild-type level begins to exert a selective cost: in addition to acceleration of multiple Clb2-dependent mitotic events, a significant frequency of catastrophic mitoses is observed. Therefore, mitotic cyclin expression levels may have evolved to an optimum expression level sufficient to buffer variability without perturbing event ordering or causing catastrophic failures.

An intriguing open question is what sets the differential Clb2-CDK thresholds for mitotic events. To address this, we need to understand the mechanisms by which various events are regulated. We observe that Clb2-CDK activity modulates an intrinsically oscillating endocycle of polarized growth. Several oscillators have been observed in the absence of CDK oscillation in budding yeast. SPB duplication, budding (which is likely the same network we observe here as polarized growth), and transcription have all been shown to cycle in the absence of Clb-CDK activity [29], [42]–[44]. Additionally, a recently-described oscillator controls periodic Cdc14 phosphatase release at stable high levels of Clb-CDK activity [22]. As proposed in that case, Clb2-CDK activity seems to phase-lock an independent polarity oscillator to the main engine of the cell cycle, limiting its execution to once-per-cycle.

For both spindle formation and spindle elongation, Clb2-CDK likely phosphorylates multiple targets to trigger the event [30], [36]–[38], [45], [46]. The full set of essential targets is not yet known, but we observe stochastic responses of these events to low Clb2 levels, suggesting the regulation of a small number of proteins. Once all essential targets are identified, it will be interesting to see how they collaborate to determine distinct thresholds for Clb-CDK activity. The answer may lie in different affinities for the kinase, multisite phosphorylation on individual proteins, or perhaps a “coincidence detector” for simultaneous phosphorylation of multiple proteins.

The observation that increasing cyclin-CDK levels are required for subsequent mitotic events suggests that mitotic cyclin-CDK is normally rate-limiting for mitosis. Indeed, we show that moderate overexpression of Clb2 from a constitutive promoter accelerates mitotic events relative to budding and DNA replication, and ultimately increases the overall frequency of the cell cycle oscillator.

Taken together, our results provide a detailed picture of how the mechanistically diverse events of mitosis can be controlled by the accumulation of a single cyclin-CDK complex. As cyclin levels rise above discrete response thresholds, events are triggered sequentially, producing order and timing separation. This is reminiscent of the induction of distinct cell fates by spatial morphogen gradients, for example in *C. elegans* vulval development [47]. In the cell cycle, the gradient is temporal, effecting qualitatively distinct cell cycle fates within a single cell as its levels traverse critical thresholds.

Moderate premature cyclin-CDK accumulation accelerates cell cycle frequency, without affecting the ordering of cell cycle events. However, even very moderate acceleration over the wild-type rate leads to a cost in catastrophic premature mitoses. Thus, mitotic cyclin expression seems to be tightly tuned to balance efficiency of event execution with a low rate of mitotic failure. We expect that the levels of similar regulators in other systems will also be optimized in this way.

## Materials and Methods

### Yeast Strains and Plasmids

Standard mating and transformation methods were used for construction of all strains. All strains used were derived from W303. To replace endogenous *CLB2* and *CLB2-YFP* with single copy alleles driven by the *GALL* promoter, a plasmid, pCL3, was used, consisting of the *GALL* promoter driving a truncated *CLB2* allele in which 783 nucleotides have been excised by MfeI digestion. For all strains and plasmids used, see Tables S1 and S2, respectively.

### Timecourses

For alpha factor timecourses, strains containing a *bar1Δ* mutation were arrested with 10 nM alpha-factor for 120′ at 30°C. Alpha-factor was removed by 3 cold washes. For Clb2-YFP pulsing, strains were grown to log phase in Synthetic Complete media without methionine +2% dextrose (SCD-Met) containing 5 mM deoxycorticosterone (DOC). DOC was removed by three cold washes in SCD-Met. After 2 hours, 0.4 g/L Met was added. 1 hour later, cells were pulsed with 5 mM DOC for variable times (5-15′). DOC was removed by 3 cold washes with SCD+Met. For timelapse imaging, cells were plated as described below on SCD+Met media. For anaphase timecourses, cells were left at 30°C for 2 hours, then methionine was removed by vacuum filtration, and cells were plated for timelapse microscopy (see below) on SCD-Met media.

### Immunoblotting and Kinase Assays

Western blotting was performed using standard methods. The following antibody concentrations were used: anti-Pgk1, 1∶10,000 (Invitrogen); anti-Clb2, 1∶10,000 (Covance); HRP-conjugated anti-rabbit and anti-mouse secondaries, 1∶5,000 (GE). Blots were imaged with a Fujifilm LAS-3000 imager. Kinase assays were performed essentially as described [48], with the following modifications: 50 mM NaF, and 1 mM sodium-orthovanadate were also added to inhibit phosphatase activity, cells were broken with a FastPrep bead beater (Thermo Scientific; 2×20 s at setting 5, with 1′ rest on ice in between), and anti-Clb2 antibody was used at a 1∶700 dilution (Covance).

### Timelapse Microscopy and Fixed Cell Imaging

Timelapse imaging was carried out using a Leica DMI6000B inverted fluorescence microscope with a 63X NA1.4 oil objective. Objective and stage were heated to 30°C. Samples were mounted on agar slabs containing appropriate media and imaged with 3′ temporal resolution. Image acquisition and processing were carried out with custom Matlab software described previously [49]. To enhance signal intensity, 2×2 binning of CCD pixels was used. Image intensity calibration beads were used to correct for day-to-day variations in lamp intensity (InSpeck Green, 2.5 µm, ∼0.3% relative intensity; Molecular Probes). Htb2-mCherry signal was used as a nuclear mask to determine mean nuclear Clb2-YFP intensity. Clb2-YFP took up to 45′ to fully mature; Clb2-YFP nuclear intensity was determined by averaging the values obtained from at least five frames after this point. For anaphase movies, Clb2-YFP was fully mature at the beginning of the movie. The resulting Clb2-YFP nuclear intensity values were normalized to the intensity of the calibration beads, background subtracted (using the mean nuclear intensity from cycling cells without YFP-labeled Clb2), and normalized to the mean of a population of synchronized cells containing peak Clb2-YFP levels. Spindle formation and elongation were scored by eye from Tub1-CFP signal. Due to variation in the focal plane of the spindle, it took up to 3 frames (9′) to definitively score spindle formation. Following validation of accuracy using Spa2-GFP localization and Matlab-based bud length calculation, growth depolarization was scored by eye from the rate of increase of bud length.

For fixed cell imaging, samples were collected by centrifugation, washed with water, and incubated with formaldehyde fixative (4% paraformaldehyde, 3.4% sucrose, 100 mM KPO_4_ pH 7.5, 100 µM MgCl_2_) at room temperature for 10′. Cells were spun down and washed 2 times with sorbitol-phosphate buffer (1.2 M sorbitol, 100 mM KPO_4_ pH 7.5, 100 µM MgCl_2_) before resuspension in 50 µl sorbitol-phosphate buffer. Fixed cells were stored at 4°C and imaged within 24 hours using a Zeiss Axioplan2 inverted fluorescent microscope with a 63X NA1.4 Plan Apo oil objective. Images were acquired using OpenLab software (Improvision) and microscope calibration beads were used as described above to correct for intensity variations between imaging sessions. Images comprised z-stacks of layers 0.5 µm apart (YFP and mCherry, 3 layers; CFP, 5 layers). Clb2-YFP signal intensity and spindle state were determined using custom Matlab software developed by the authors. Briefly, background was subtracted from raw YFP images and nuclei demarcated by a thresholding function performed on raw mCherry images. Mean YFP intensity in each nucleus was calculated and normalized to the mean of a population of cells synchronized at the time of peak Clb2-YFP expression. The number of distinct Spc29-CFP signals in each cell body was automatically determined; 1 signal was scored as no spindle, 2 signals were scored as a spindle.

To score growth depolarization, cells were fixed as described above for 1 hour, incubated with rhodamine-phalloidin (Invitrogen) for 1 hour in darkness, then washed 5 times with sorbitol-phosphate buffer. Polarization index was determined using Matlab by the following method: the bud contour (edge) was traced, and the mean fluorescent intensity of the middle third of the contour (corresponding to the bud tip) was divided by the mean fluorescent intensity of the remaining two-thirds. Values around 1 indicate uniform actin distribution; values greater than 1 indicate tip-biased localization.

### Modeling Spindle Formation at Fixed Clb2 Levels

The ODE describing spindle formation was taken from [35]:
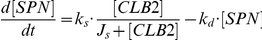
with the associated rate constants:

k_s_ = 0.1

J_s_ = 0.14

k_d_ = 0.06

This ODE was simulated with Matlab for various fixed levels of [*CLB2*] corresponding to the stable Clb2-YFP levels provided experimentally (expressed as a fraction of peak Clb2).

### ODE Model for Clb2-CDK Phase-Locking of Polarization Cycle

We adapted a simple mathematical phase-locking model from [22]. The treatment of coupling between oscillators is as described in [50], modified for one-way phase-locking. Briefly, a Clb2-CDK oscillator (ϕ) oscillates with intrinsic frequency (f(ϕ), arbitrarily set to 1). A peripheral oscillator controlling polarized growth (ψ) oscillates with an intrinsic frequency f(ψ), expressed as a fraction of f(ϕ). This peripheral cycle is sensitive to forcing by ϕ when sin(ψ) > Z_lim_. For example, Z_lim_ = 1 indicates that ψ is never sensitive; −1, always sensitive; 0, sensitive half the time. C is a coupling variable characterizing the strength of ϕ's effect on f(ψ). In this case, ϕ is a fixed quantity corresponding to the fixed Clb2 levels we provide experimentally, expressed as a fraction of the peak. 
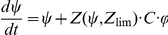
where
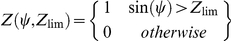



Parameter values were estimated from experiments. f(ψ) is estimated as the frequency of budding in the absence of Clb2-CDK activity (1/80′, see Figure 2) divided by the total cell cycle frequency, f(ϕ) (1/100′), giving 1.25. We assume that the budding cycle is sensitive during its polarized (‘on’) portion, which is half the total cycle time, giving Z_lim_ = 0. C = 2 was arbitrarily determined as it gave us a response to fixed levels of ϕ similar to what we observed experimentally (Fig. 2, Fig. S4). Simulation was carried out with Matlab.

### Assaying the Dose Requirement for Spindle Formation in CLB2^WT^ Cells

*clb1,3,4Δ CLB2-YFP MET3:CDC20* cells carrying a *bar1Δ* mutation were synchronized with 10 nM alpha factor and released into SCD-Met. At 45′, 50′, or 55′ post-release, 200 µg/mL cycloheximide was added to stop protein translation (and thus further Clb2-YFP accumulation). One hour later, cells were fixed for imaging. Mean nuclear Clb2-YFP was measured using custom Matlab software as before (background subtracted and normalized to peak Clb2-YFP), and spindle state was assayed by eye from Tub1-CFP signal.

### Stochastic Model for Event Triggering by Multiple Phosphorylations

We assumed that an event is triggered by phosphorylation of a discrete number of individual targets, each of which occurs independently, as should be the case with distinct biological targets (*i.e.* separate proteins). The probability that a target is phosphorylated is described by a Poisson distribution.

The probability of a given number of new phosphorylation events, κ, is given by:
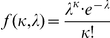
where λ represents the activity of the kinase and is directly proportional to fixed Clb2 level.

The probability of dephosphorylation events is similarly described, where λ represents the activity of the phosphatase.

In each time increment, the number of targets phosphorylated is adjusted by adding new phosphorylations and subtracting new dephosphorylations, until either the number of targets phosphorylated reaches the threshold (triggering the process, in the simulation) or a time limit is reached. In the example shown, the threshold was set to three. To generate the illustrative plot, the time the event was triggered was plotted for 50 trials at each fixed Clb2 level.

## Supporting Information

Figure S1 **Timing difference between** ***CLB^WT^*** **and** ***clb1,3,4Δ*** **cells.** Cycling cells of the indicated genotypes and containing GFP-labeled tubulin (Tub1-GFP) were observed by timelapse microscopy. Budding and anaphase were scored by eye and the resulting intervals plotted as shown. Mean ± s.d. is shown inset for each distribution.
(TIF)

Figure S2 **Swe1 inhibits Clb2-CDK in early mitosis.** **A** Cells of the experimental strain, with or without *SWE1* (blue and red, respectively), were blocked by Clb2 depletion (DOC washout) and given a pulse of Clb2-YFP. Clb2-YFP protein and associated kinase activity were assayed at indicated timepoints following the pulse. Quantification of kinase activity (normalized to Pgk1 protein level) is shown at right. **B** WT (blue) and *swe1Δ* (red) strains were synchronized with alpha factor and released. Clb2-YFP protein and associated kinase activity were assayed. Quantification of kinase activity (normalized to Pgk1 protein level) is shown at right.
(TIF)

Figure S3 **Separation of growth depolarization, spindle formation in cycling cells.** *clb1,3,4Δ* cells were synchronized with alpha factor and released. Samples were fixed at the indicated times and analyzed for growth polarization (blue) and spindle formation (red). Polarization index ∼1 indicates depolarized growth; >1 indicates polarized growth.
(TIF)

Figure S4 **A model for phase-locking of an independent growth polarity oscillator by Clb2-CDK activity.** **A** Schematic of coupled Clb-CDK and polarization oscillators. Clb-CDK can force the polarization oscillator during its sensitive period (shown here in red) to phase-lock the two oscillators. Pol., polarized growth; Depol., depolarized growth. **B** Left: uncoupled oscillations of budding (red) and Clb2-CDK (blue) in the absence of phase-locking. Right: coupled oscillations driven by phase-locking of budding cycle by Clb2 cycle. **C** Response of budding cycle frequency to fixed levels of Clb2 (coupling  =  2).
(TIF)

Figure S5 **Clb2-YFP requirement for spindle formation is independent of detection method.** **A** Cells containing *SPC29-CFP* were pulsed with Clb2-YFP as described previously, and fixed after 60′. Clb2-YFP nuclear intensity and spindle formation (defined as two separated dots of Spc29-CFP) were assayed from images of fixed cells. The distribution of cells that formed spindles (red) is compared to data from timelapse imaging shown in Figure 3 using Tub1-CFP (blue). N  =  228. **C** *clb1,3,4Δ CLB2-YFP* cells were synchronized with alpha factor and cycloheximide was added to stop protein synthesis at variable timepoints after release (45-55′). One hour later, cells were fixed and Clb2-YFP nuclear intensity and spindle status (scored with Tub1-CFP) were assayed. The fraction of cells with spindles is shown in red as a function of Clb2-YFP level, compared to the experimental data from timelapse imaging (see Figure 3) shown in blue.
(TIF)

Figure S6 **A stochastic model for event triggering by multiple phosphorylations.** The results of a simulation requiring three independent phosphorylations to trigger an event are shown. The time of the event is plotted as a function of fixed Clb2 level. The results of 50 simulations are shown for each Clb2 level.
(TIF)

Table S1 DOC

Table S2 DOC

Video S1 Spa2-GFP in a cell of the experimental strain arrested *without* induction of Clb2-YFP. 1 frame  =  3′.
(AVI)

Video S2 Spindle formation followed with Tub1-CFP in a cell of the experimental strain following a pulse of Clb2-YFP. 1 frame  =  3′.
(AVI)

Video S3 Spindle elongation followed with Tub1-CFP in a cell of the experimental strain following a pulse of Clb2-YFP and *CDC20* re-expression. 1 frame  =  3′.
(AVI)

Video S4 Tub1-CFP in a cell of the experimental strain that does not undergo spindle elongation following a pulse of Clb2-YFP and *CDC20* re-expression. 1 frame  =  3′.
(AVI)

Video S5 Premature anaphase phenotype of *GALL:CLB2* strain cycling in galactose. Green: Tub1-GFP; red: Htb2-mCherry. 1 frame  =  3′.
(AVI)

## References

[1] Loog M, Morgan DO (2005). Cyclin specificity in the phosphorylation of cyclin-dependent kinase substrates.. Nature.

[2] Bloom J, Cross FR (2007). Multiple levels of cyclin specificity in cell-cycle control.. Nat Rev Mol Cell Biol.

[3] Fitch I, Dahmann C, Surana U, Amon A, Nasmyth K (1992). Characterization of four B-type cyclin genes of the budding yeast Saccharomyces cerevisiae.. Mol Biol Cell.

[4] Richardson H, Lew DJ, Henze M, Sugimoto K, Reed SI (1992). Cyclin-B homologs in *Saccharomyces cerevisiae* function in S phase and in G2.. Genes Dev.

[5] Archambault V, Buchler NE, Wilmes GM, Jacobson MD, Cross FR (2005). Two-Faced Cyclins with Eyes on the Targets.. Cell Cycle.

[6] Kellis M, Birren BW, Lander ES (2004). Proof and evolutionary analysis of ancient genome duplication in the yeast Saccharomyces cerevisiae.. Nature.

[7] Hartwell LH, Weinert TA (1989). Checkpoints: controls that ensure the order of cell cycle events.. Science.

[8] Weinert TA, Kiser GL, Hartwell LH (1994). Mitotic checkpoint genes in budding yeast and the dependence of mitosis on DNA replication and repair.. Genes Dev.

[9] Cross FR, Archambault V, Miller M, Klovstad M (2002). Testing a Mathematical Model of the Yeast Cell Cycle.. Mol Biol Cell.

[10] Pringle JR, Hartwell LH, Strathern JN, Jones EW, Broach JR (1981). The *Saccharomyces cerevisiae* Cell Cycle.. The Molecular Biology of the Yeast *Saccharomyces*.

[11] Stern B, Nurse P (1996). A Quantitative Model for the cdc2 Control of S Phase and Mitosis in Fission Yeast.. Trends in Genetics.

[12] Coudreuse D, Nurse P (2010). Driving the cell cycle with a minimal CDK control network.. Nature.

[13] Gavet O, Pines J (2010). Progressive activation of CyclinB1-Cdk1 coordinates entry to mitosis.. Dev Cell.

[14] Deibler RW, Kirschner MW (2010). Quantitative reconstruction of mitotic CDK1 activation in somatic cell extracts.. Mol Cell.

[15] Amon A, Surana U, Muroff I, Nasmyth K (1992). Regulation of p34CDC28 tyrosine phosphorylation is not required for entry into mitosis in S. cerevisiae.. Nature.

[16] Mumberg D, Muller R, Funk M (1994). Regulatable promoters of Saccharomyces cerevisiae: comparison of transcriptional activity and their use for heterologous expression.. Nucleic Acids Research.

[17] West RW, Yocum RR, Ptashne M (1984). Saccharomyces cerevisiae GAL1-GAL10 divergent promoter region: location and function of the upstream activating sequence UASG.. Mol Cell Biol.

[18] Picard D, Picard D (1999). Regulation of heterologous proteins by fusion to a hormone binding domain.. Nuclear receptors: a practical approach.

[19] Drapkin BJ, Lu Y, Procko AL, Timney BL, Cross FR (2009). Analysis of the mitotic exit control system using locked levels of stable mitotic cyclin.. Mol Syst Biol.

[20] Hood JK, Hwang WW, Silver PA (2001). The Saccharomyces cerevisiae cyclin Clb2p is targeted to multiple subcellular locations by cis- and trans-acting determinants.. J Cell Sci.

[21] Jorgensen P, Edgington NP, Schneider BL, Rupes I, Tyers M (2007). The size of the nucleus increases as yeast cells grow.. Mol Biol Cell.

[22] Lu Y, Cross FR (2010). Periodic cyclin-Cdk activity entrains an autonomous Cdc14 release oscillator.. Cell.

[23] Keaton MA, Lew DJ (2006). Eavesdropping on the cytoskeleton: progress and controversy in the yeast morphogenesis checkpoint.. Current Opinion in Microbiology.

[24] Amon A, Tyers M, Futcher B, Nasmyth K (1993). Mechanisms that help the yeast cell cycle clock tick: G2 cyclins transcriptionally activate G2 cyclins and repress G1 cyclins.. Cell.

[25] Verma R, Annan RS, Huddleston MJ, Carr SA, Reynard G (1997). Phosphorylation of Sic1p by G1 Cdk required for its degradation and entry into S phase.. Science.

[26] Schwob E, Bohm T, Mendenhall MD, Nasmyth K (1994). The B-type cyclin kinase inhibitor p40SIC1 controls the G1 to S transition in S. cerevisiae.. Cell.

[27] Lew DJ, Reed SI (1993). Morphogenesis in the Yeast Cell Cycle: Regulation by Cdc28 and Cyclins.. The Journal of Cell Biology.

[28] Snyder M (1989). The SPA2 protein of yeast localizes to sites of cell growth.. J Cell Biol.

[29] Haase SB, Reed SI (1999). Evidence that a free-running oscillator drives G1 events in the budding yeast cell cycle.. Nature.

[30] Anderson VE, Prudden J, Prochnik S, Giddings THJ, Hardwick KG (2007). Novel *sfi1* alleles uncover additional functions for Sfi1p in bipolar spindle assembly and function.. Mol Biol Cell.

[31] Li S, Sandercock AM, Conduit P, Robinson CV, Williams RL (2006). Structural role of Sfi1p-centrin filaments in budding yeast spindle pole body duplication.. J Cell Biol.

[32] Surana U, Robitsch H, Price C, Schuster T, Fitch I (1991). The role of CDC28 and cyclins during mitosis in the budding yeast S. cerevisiae.. Cell.

[33] Harvey SL, Kellogg DR (2003). Conservation of Mechanisms Controlling Entry into Mitosis: Budding Yeast Wee1 Delays Entry into Mitosis and Is Required for Cell Size Control.. Current Biology.

[34] McNulty JJ, Lew DJ (2005). Swe1p Responds to Cytoskeletal Perturbation, Not Bud Size, in S. cerevisiae.. Current Biology.

[35] Chen KC, Calzone L, Csikasz-Nagy A, Cross FR, Novak B (2004). Integrative analysis of cell cycle control in budding yeast.. Mol Biol Cell.

[36] Rudner AD, Hardwick KG, Murray AW (2000). Cdc28 activates exit from mitosis in budding yeast.. JCB.

[37] Rudner AD, Murray AW (2000). Phosphorylation by Cdc28 activates the Cdc20-dependent activity of the Anaphase-Promoting Complex.. JCB.

[38] Rahal R, Amon A (2008). Mitotic CDKs control the metaphase-anaphase transition and trigger spindle elongation.. Genes Dev.

[39] Cross FR (1995). Starting the cell cycle: what′s the point?. Curr Opin Cell Biol.

[40] Surana U, Amon A, Dowzer C, McGrew J, Byers B (1993). Destruction of the CDC28/CLB mitotic kinase is not required for the metaphase to anaphase transition in budding yeast.. EMBO J.

[41] Ghaemmaghami S, Huh W, Bower K, Howson RW, Belle A (2003). Global analysis of protein expression in yeast.. Nature.

[42] Hartwell LH (1971). Genetic control of the cell division cycle in yeast. II. Genes controlling DNA replication and its initiation.. J Mol Biol.

[43] Haase SB, Winey M, Reed SI (2001). Multi-step control of spindle pole body duplication by cyclin-dependent kinase.. Nat Cell Biol.

[44] Orlando DA, Lin CY, Bernard A, Wang JY, Socolar JES (2008). Global control of cell-cycle transcription by coupled CDK and network oscillators.. Nature.

[45] Crasta K, Huang P, Morgan G, Winey M, Surana U (2006). Cdk1 regulates centrosome separation by restraining proteolysis of microtubule-associated proteins.. EMBO.

[46] Chee MK, Haase SB (2010). B-cyclin/CDKs regulate mitotic spindle assembly by phosphorylating kinesins-5 in budding yeast.. PLoS Genet.

[47] Katz WS, Hill RJ, Clandinin TR, Sternberg PW (1995). Different levels of the C. elegans growth factor LIN-3 promote distinct vulval precursor fates.. Cell.

[48] Levine K, Huang K, Cross FR (1996). Saccharomyces cerevisiae G1 cyclins differ in their intrinsic functional specificities.. Mol Cell Biol.

[49] Charvin G, Cross FR, Siggia ED (2008). A microfluidic device for temporally controlled gene expression and long-term fluorescent imaging in unperturbed dividing yeast cells.. PLoS One.

[50] Strogatz SH (1994). Nonlinear Dynamics and Chaos..

