# Supplementary material for: Rising Cyclin-CDK Levels Order Cell Cycle Events
Source: PLoS One. 2011 Jun 10;6(6):e20788. doi: 10.1371/journal.pone.0020788 (PMC3112166; doi:10.1371/journal.pone.0020788)
Supplement: Table S1 — DOC [file pone.0020788.s007.doc]

Table S1. Yeast strains used in this study.

| **Strain** | **MAT** | **Genotype** |
| --- | --- | --- |
| FM224 | a | CLB2-YFP::HIS5 CHS2-CFP::HIS5 (gift from Foong May Yeong) |
| CO172 | a | clb1 clb2::GALL:CLB2-YFP::URA3::HIS3 clb3::TRP1 clb4::his3::kanMX cdc20::MET:HA3-CDC20::TRP1 ADH1:GAL4rMR::HIS5 TUB1-CFP::HIS3 HTB2-mCherry::HIS3 MYO1-mCherry::HIS3 ADE2 |
| CO203 | a | clb1 clb2::GALL:CLB2-YFP::URA3::HIS3 clb3::TRP1 clb4::his3::kanMX cdc20::MET:HA3-CDC20::TRP1 ADH1:GAL4rMR::HIS5 TUB1-CFP::HIS3 HTB2-mCherry::HIS3 MYO1-mCherry::HIS3 LEU2::SPA2-GFP ADE2 |
| CO207 | a | clb1 clb2::GALL:CLB2-YFP::URA3::HIS3 clb3::TRP1 clb4::his3::kanMX cdc20::MET:HA3-CDC20::TRP1 ADH1:GAL4rMR::HIS5 SPC29-CFP::kanMX HTB2-mCherry::HIS3 ADE2 |
| CO239 | a | clb1 clb2::GALL:CLB2-YFP::URA3::HIS3 clb3::TRP1 clb4::his3::kanMX ADH1:GAL4rMR::HIS5 cdc20::MET:HA3-CDC20::TRP1 swe1::URA3 TUB1-CFP::TRP1 HTB2-mCherry::HIS3 ADE2 |
| CO240-1 | α | clb1 clb3::TRP1 clb4::his3::kanMX cdc20::MET:HA3-CDC20::TRP1 TUB1-GFP::HIS3 ADE2 |
| CO240-2 | α | cdc20::MET:HA3-CDC20::TRP1 TUB1-GFP::HIS3 ADE2 |
| CO243 | a | bar1 clb1 CLB2-YFP::HIS5 clb3::TRP1 clb4::his3::kanMX cdc20::MET:HA3-CDC20::TRP1 MYO1-mCherry::HIS5 TUB1-mCherry::URA3 TUB1-CFP::TRP1 ADE2 |
| CO303-5 | a | bar1 clb1::URA3 clb3::TRP1 clb4::HIS5 TUB1-GFP::HIS3 ADE2 |
| CO303-8 | a | bar1 clb1::URA3 clb3::TRP1 clb4::HIS5 swe1::TRP1 TUB1-GFP::HIS3 ADE2 |
| CO324 | a | bar1 clb2::GALL:CLB2::URA3 TUB1-GFP::HIS3 ADE2 |
| CO325 | a | bar1 TUB1-GFP::HIS3 URA3 ADE2 |
| CO331 | a | clb2::GALL:CLB2::URA3 TUB1-GFP::HIS3 HTB2-mCherry::HIS3 ADE2 |
