# Supplementary material for: Rising Cyclin-CDK Levels Order Cell Cycle Events
Source: PLoS One. 2011 Jun 10;6(6):e20788. doi: 10.1371/journal.pone.0020788 (PMC3112166; doi:10.1371/journal.pone.0020788)
Supplement: Table S2 — DOC [file pone.0020788.s008.doc]

Table S2. Plasmids used in this study.

| **Plasmid** | **Description** | **Source** |
| --- | --- | --- |
| pMR5125 | pRS416 – SPA2-GFP | M. Rose |
| p313GAL4rMR | pRS313 – ADH1pr-GAL4rMR | N. Buchler |
| pCO3 | pRS406 – GALL-clb2∆ | this study |
| pCO5 | pRS403 – ADH1pr-GAL4rMR | this study |
| pCO30 | pRS405 – SPA2-GFP | this study |
